# Severe Hyperkalemia: Can the Electrocardiogram Risk Stratify for Short-term Adverse Events?

**DOI:** 10.5811/westjem.2017.6.33033

**Published:** 2017-07-10

**Authors:** Nicole Durfey, Brian Lehnhof, Andrew Bergeson, Shayla N.M. Durfey, Victoria Leytin, Kristina McAteer, Eric Schwam, Justin Valiquet

**Affiliations:** * Kent Hospital, Department of Emergency Medicine, Warwick, Rhode Island; † The Warren Alpert Medical School of Brown University, Providence, Rhode Island; ‡ The Warren Alpert Medical School of Brown University, Department of Emergency Medicine, Providence, Rhode Island

## Abstract

**Introduction:**

The electrocardiogram (ECG) is often used to identify which hyperkalemic patients are at risk for adverse events. However, there is a paucity of evidence to support this practice. This study analyzes the association between specific hyperkalemic ECG abnormalities and the development of short-term adverse events in patients with severe hyperkalemia.

**Methods:**

We collected records of all adult patients with potassium (K+) ≥6.5 mEq/L in the hospital laboratory database from August 15, 2010, through January 30, 2015. A chart review identified patient demographics, concurrent laboratory values, ECG within one hour of K+ measurement, treatments and occurrence of adverse events within six hours of ECG. We defined adverse events as symptomatic bradycardia, ventricular tachycardia, ventricular fibrillation, cardiopulmonary resuscitation (CPR) and/or death. Two emergency physicians blinded to study objective independently examined each ECG for rate, rhythm, peaked T wave, PR interval duration and QRS complex duration. Relative risk was calculated to determine the association between specific hyperkalemic ECG abnormalities and short-term adverse events.

**Results:**

We included a total of 188 patients with severe hyperkalemia in the final study group. Adverse events occurred within six hours in 28 patients (15%): symptomatic bradycardia (n=22), death (n=4), ventricular tachycardia (n=2) and CPR (n=2). All adverse events occurred prior to treatment with calcium and all but one occurred prior to K^+^-lowering intervention. All patients who had a short-term adverse event had a preceding ECG that demonstrated at least one hyperkalemic abnormality (100%, 95% confidence interval [CI] [85.7–100%]). An increased likelihood of short-term adverse event was found for hyperkalemic patients whose ECG demonstrated QRS prolongation (relative risk [RR] 4.74, 95% CI [2.01–11.15]), bradycardia (HR<50) (RR 12.29, 95%CI [6.69–22.57]), and/or junctional rhythm (RR 7.46, 95%CI 5.28–11.13). There was no statistically significant correlation between peaked T waves and short-term adverse events (RR 0.77, 95% CI [0.35–1.70]).

**Conclusion:**

Our findings support the use of the ECG to risk stratify patients with severe hyperkalemia for short-term adverse events.

## INTRODUCTION

Severe hyperkalemia can lead to lethal cardiac dysrhythmias. Hyperkalemia produces cardiotoxicity through early depolarization of the cell membrane, slowing of ventricular conduction and decreased durations of the action potential. 1,2 These changes at the cellular level correlate with the electrocardiogram (ECG) manifestations of hyperkalemia. Traditional teaching describes the sequential appearance of ECG abnormalities seen with rising potassium (K^+^) levels as follows: peaked T waves, PR interval prolongation, QRS prolongation, loss of P wave, escape rhythms, “sine wave” configuration, ventricular fibrillation, and pulseless activity or asystole^1^,^2^ The presence or absence of these ECG manifestations of hyperkalemia are frequently used to determine how aggressively a hyperkalemic patient is treated.^3^–^5^

However, studies have demonstrated that ECGs without any findings consistent with hyperkalemia are seen in 50–64% of patients with K^+^ ≥ 6.5 mEq/L.^6^–^8^ Cases of patients with extreme hyperkalemia (10.1–10.3 mEq/L) and normal ECGs have also been reported.^9^,^10^ Furthermore, dysrhythmia and cardiac arrest have been reported in hyperkalemic patients without preceding peaked T waves.^11^ The role of the ECG in the management of hyperkalemia has thus been increasingly called into question.^2^,^7^,^11^–^14^ Leading FOAMed (free online open-access medical education) educators have deemphasized the role of ECG in management decisions, warning that patients with relatively normal ECGs may still experience sudden hyperkalemic cardiac arrest.^12^,^14^ A recently published guideline for the management of severe hyperkalemia called for further research to both characterize the actual risk of cardiac instability in hyperkalemic patients without ECG abnormalities and to identify which hyperkalemic ECG changes are the greatest predictors of outcome.^15^

The practice of using the ECG to determine how aggressively to treat hyperkalemia assumes that ECG changes reliably occur prior to hyperkalemic dysrhythmia or cardiac arrest. While this is a widely held belief, the level of evidence needed to support this teaching does not currently exist. The objective of this study was to determine the association between specific hyperkalemic ECG abnormalities and the development of short-term adverse events in patients with severe hyperkalemia. This study could represent the first step in creating a predictive model for the risk stratification of hyperkalemic patients based on ECG changes.

## METHODS

### Study Design

This was an observational retrospective cohort study. The study received institutional review board approval, with a waiver of informed consent.

Population Health Research Capsule

What do we already know about this issue?

Hyperkalemic ECG abnormalities do not consistently occur in hyperkalemic patients. Whether the ECG identifies patients at higher risk for adverse events is unclear.

What was the research question?

What is the association between ECG abnormalities and short-term adverse events in patients with hyperkalemia?

What was the major finding of the study?

All short-term adverse events in hyperkalemic patients were preceded by ECG abnormalities.

How does this improve population health?

This study suggests that the ECG is a useful tool in the risk-stratification of hyperkalemic patients.

### Study Setting and Population

The study was performed at a suburban community hospital that supports an emergency medicine residency program. The annual emergency department (ED) census is approximately 72,000 patients. Patients are primarily adults (90%); approximately 90% are White, 4% are Hispanic, and 2% are Black.

A list of medical record numbers for all adult patients (age ≥ 18 years) with K^+^ ≥6.5 mEq/L from August 15, 2010, through January 30, 2015, was electronically generated from the hospital laboratory database. This database contains all laboratory data for ED and hospitalized patients, which ensured that all hyperkalemia values were captured.

We developed inclusion and exclusion criteria prior to data collection. Cases selected for inclusion were required to have a documented serum or plasma K^+^ of ≥6.5 mEq/L and an ECG performed within one hour of the laboratory draw. The K^+^ ≥6.5 mEq/L cutoff is considered a threshold for initiating emergency therapy, and has been used in prior publications.^2^,^16^ The ECG could be performed either during the 60 minutes prior to the acquisition of the K^+^ sample, or during the 60 minutes after the laboratory draw. When a serum and plasma K^+^ level were both obtained, the plasma level (also known as a heparinized K^+^) was used. Only one episode of hyperkalemia per patient was included in the study. We excluded recurrent episodes of hyperkalemia in the same patient.

Exclusion criteria included laboratory notation of a hemolyzed sample, platelet count ≥ 500 × 10^9^ /L, paced rhythm on ECG, and treatment for hyperkalemia prior to obtaining the ECG and laboratory sample. Hemolyzed samples were excluded because the release of K^+^ from red blood cells during hemolysis can lead to false elevation of the serum potassium. Similarly, we excluded patients with platelet count ≥ 500 × 10^9^ /L because this degree of thrombocytosis can cause pseudohyperkalemia. Treatment for hyperkalemia was defined as the administration of any of the following prior to the time the ECG was obtained and the laboratory sample was collected: calcium chloride or gluconate, sodium bicarbonate, albuterol, insulin, dextrose, sodium polystyrene sulfonate, and/or hemodialysis. Patients who received prior treatment for hyperkalemia were excluded so that the measured K^+^ level more accurately reflected the K^+^ value at the time of the ECG. We also excluded patients if they received atropine, dopamine, epinephrine, norepinephrine, or vasopressin prior to the time the ECG was obtained. We excluded these patients because of the potential of these medications to alter the ECG, such as precipitating ventricular tachycardia or masking hyperkalemic bradycardia.

### Study Protocol

We abstracted data from the electronic medical record (EMR), including ED record, admission history and physical, daily progress notes, discharge summary, and electronic medication administration record. A standardized, closed-ended electronic data collection form was used. All reviewers (AB, SD, BL, JV) were trained in the data collection rules and definitions using sample medical records. All EMRs and data collection forms of the final study group (n=188) were reviewed for accuracy by a second reviewer (ND).

The following information was abstracted from each record: (1) demographics (age, sex, race); (2) serum and plasma K^+^ levels and time obtained; (3) patient location at time of hyperkalemia (ED vs inpatient); (4) ECG and time obtained; (5) medications administered prior to obtaining ECG (including medications administered by emergency medical services in the prehospital setting) and in the six hours after the ECG; (6) laboratory values (sodium, calcium, glucose, creatinine, CO_2_, platelets) obtained on the same lab draw as the K^+^ level; (7) whether the patient was an established dialysis patient at the time of the episode of hyperkalemia; and (8) occurrence of a study-defined adverse event in the six hours after the ECG. All charts were reviewed by two reviewers for the presence or absence of an adverse event (BL, AB, JV). Disagreement was resolved by the primary investigator (ND).

We obtained a copy of the ECG performed within one hour of laboratory draw, and prior to treatment. When available, we also obtained a copy of the most recent previous ECG to serve as a baseline. K^+^ level was confirmed to be <5.0 mEq/L at time of previous ECG.

We created a separate document containing only the initial ECG, previous ECG (when available) and an event identifier. A second standardized, closed-ended electronic data collection form was used to review all ECGs. All ECGs were reviewed by two experienced board-certified emergency physicians (VL, ES). Both reviewers were blinded to the objectives and methods of the study, the potassium value, associated medical history, clinical information, and all other data collected for the patient. The ECG reviewers were also blinded to the formal interpretation documented by the attending cardiologist, as well as each other’s readings. The reviewers independently examined each ECG for rate, rhythm, peaked T wave, PR interval duration, QRS wave duration, and type of intraventricular conduction delay (if present). If the reviewer agreed with the computer-generated values of PR interval and QRS wave duration (in milliseconds), then we used the computer-generated values. To keep the ECG reviewers blinded to the study objective, additional data that did not pertain to the objective of the study (left ventricular hypertrophy, ST elevation, ST depression and/or T wave inversion) were included in the data collection form.

We categorized the ECG as “PR prolongation” if the PR interval was >200 ms, and either there was no previous ECG for comparison or the PR interval was <200 ms on the previous ECG. If the previous ECG had a PR interval >200 ms, then the ECG was categorized as “PR prolongation” if the current PR interval was longer than the previous PR interval. Similarly, we categorized the ECG as “QRS prolongation” if the QRS duration was >110 ms, and either there was no previous ECG for comparison or the QRS duration was <110 ms on the previous ECG. If the previous ECG had a QRS duration of >110 ms, then the ECG was categorized as “QRS prolongation” if the current QRS duration was longer than the previous QRS duration. In the scenario where the ECG reviewers disagreed on the rhythm, type of intraventricular conduction delay, or whether T waves were peaked or not, then we used the attending cardiologist reading.

### Outcomes

We categorized ECGs as having “any abnormality suggestive of hyperkalemia” if one or more of the following were present: (1) peaked T waves; (2) PR prolongation; (3) QRS prolongation; (4) bradycardia (HR<50 bpm); (5) 2^nd^ or 3^rd^ degree heart block; (6) junctional rhythm; (7) ventricular escape rhythm; or (8) ventricular tachycardia.

The presence or absence of an adverse event within six hours of the laboratory measurement of a K^+^ ≥6.5 mEq/L (regardless of treatment status) was determined. We defined an adverse event as symptomatic bradycardia, ventricular tachycardia, ventricular fibrillation, cardiopulmonary resuscitation (CPR) and/or death. Symptomatic bradycardia was defined as bradycardia requiring treatment with calcium chloride, calcium gluconate, atropine, epinephrine, dopamine and/or pacing for symptoms of hypotension, syncope, chest pain, dyspnea and/or altered mental status. Calcium chloride or gluconate administered solely for asymptomatic bradycardia, an abnormal ECG or high potassium value was not recorded as an adverse event.

### Data Analysis

For the association of short-term adverse events with specific ECG abnormalities, we used the Pearson chi-square statistic. Fisher’s exact test was used for analysis involving less than five events. We analyzed each variable separately and calculated relative risk (RR). The relationship of the K^+^ value to an ECG with “any abnormality suggestive of hyperkalemia” and to short-term adverse events was determined using binary logistic regression. We included K^+^ as a continuous variable in this model. The kappa statistic was calculated to evaluate the level of agreement between ECG reviewers for ECG variables, as well as for the level of agreement between reviewers for adverse events. We ran tests with SPSS (version 22; IBM Corp, Armonk, NY).

## RESULTS

**Figure 1:**
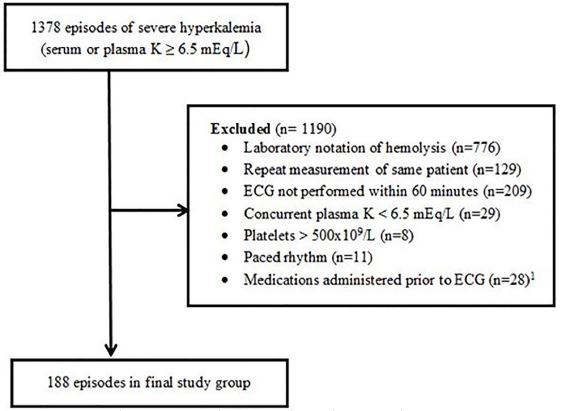
Flow diagram for inclusion in a study examining the association between electrocardiographic (ECG) abnormalities and short-term adverse events in patients with hyperkalemia. ^1^ Atropine, dopamine, epinephrine, norepinephrine, vasopressin, calcium chloride, calcium gluconate, sodium bicarbonate, albuterol, insulin, and/or sodium polystyrene sulfonate. ^2^ No patients received diuretics, dobutamine, isoproterenol or milrinone.

The final study group included 188 episodes of severe hyperkalemia (Figure 1). The majority of episodes (n=176, 94%) occurred in the ED. Mean patient age was 68 years (range 21–94 years), 54% were male, and 94% were White (Table 1). All patients had abnormal kidney function. Half of the patients had an estimated glomerular filtration rate of less than 15 mL/min/1.73m^2^. Established hemodialysis patients represented 32 (17%) of the 188 patients. Established hemodialysis patients and non-dialysis patients had no significant difference in the frequency of ”any ECG abnormality suggestive of hyperkalemia” (RR 1.02, 95% CI [0.81–1.29]) or of short term adverse events ((RR 1.34, 95% CI [0.58–3.06]).

**Figure 2:**
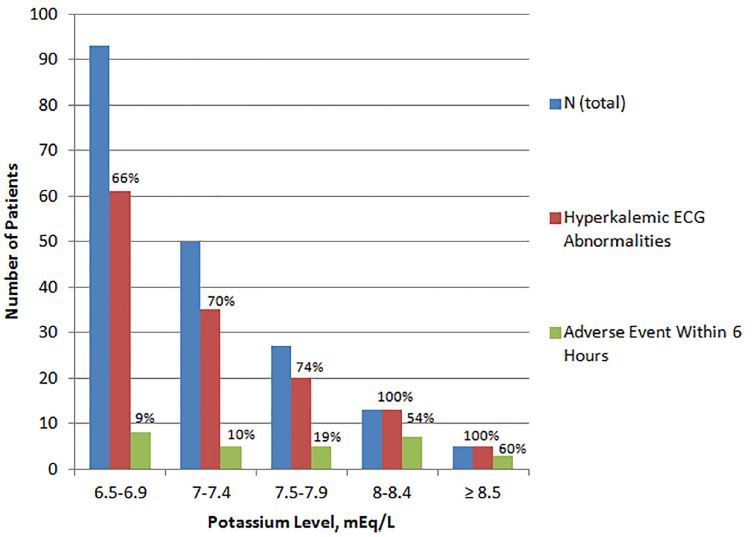
Hyperkalemic electrocardiographic (ECG) abnormalities and six-hour adverse events in patients with severe hyperkalemia (K^+^≥6.5 mEq/L). Potassium level was predictive of having “any ECG abnormality suggestive of hyperkalemia” (OR 2.71, 95% CI [1.31–5.59]) and of adverse event within six hours (OR 3.35, 95% CI [1.72–6.53]). We have not adjusted for potentially relevant covariates.

The mean serum K^+^ level was 7.1 mEq/L (SD=0.6mEq/L). The distribution of K^+^ values is presented in Figure 2. Potassium levels ranged from 6.5–9.3 mEq/L. A plasma K^+^ level was obtained on the same laboratory draw as the serum K^+^ level in 96 episodes (51%).

The ECG findings are characterized in Table 2. The mean time between the ECG and K^+^ lab draw was 18 minutes (SD=14 minutes). Previous ECGs were available for comparison in 123 episodes (65%). There was no statistical difference between the frequency of ”any ECG abnormality suggestive of hyperkalemia” in patients with previous ECG available and patients who did not have a previous ECG available (RR 0.92, 95% CI [0.74–1.10]). The RR for adverse events in patients with previous ECG available was comparable to those for the full study population (Appendix).

A total of 134 episodes (71%, 95% CI [64.4%–77.3%]) had “any ECG abnormality suggestive of hyperkalemia,” with the two most common findings being QRS prolongation (43%, 95% CI [36.7%–50.8%]) and peaked T waves (30%, 95% CI [24.1%–37.2%]). More than half (n=77, 57%) had only a single hyperkalemic ECG abnormality. Multiple hyperkalemic ECG abnormalities were present in the other 57 episodes (43%), with the most frequent combination of findings being QRS prolongation with peaked T waves.

We identified 28 patients (15%, 95% CI [10.4%–20.7%]) as having had an adverse event within six hours of the measurement of hyperkalemia. The mean K^+^ value in patients with an adverse event was 7.5 mEq/L (SD=0.7). Adverse events included symptomatic bradycardia (n=22, 12%), ventricular tachycardia (VT) (n=2, 1%), cardiopulmonary resuscitation (CPR) (n=2, 1%) and death (n=4, 2%). Two patients experienced more than one adverse event. One patient with VT survived after a brief period of CPR. Another patient died after CPR for pulseless electrical activity. Three deaths occurred in patients who did not receive CPR as they were “do not resuscitate.” All patients with symptomatic bradycardia or VT improved after treatment with calcium.

The median time from the ECG to the adverse event was 47 minutes. Adverse events occurred either prior to the laboratory notification of hyperkalemia (n=16, 59%) or shortly after the laboratory notification of hyperkalemia (mean 36 min; SD 19 min). All adverse events occurred prior to treatment with calcium, and all but one occurred prior to K^+^-lowering intervention. The majority of patients (n=177, 95%) received treatment within the six-hour period. The median time from ECG to treatment was 85 minutes. There was no significant difference in time to treatment between patients with or without adverse event, nor for each particular ECG finding. The rate of adverse events after treatment with calcium was 0% (95% CI [0–4.0%]) and after K^+^-lowering intervention was 0.7% (95% CI [ <0.01%–3.5%]).

All of the 28 patients with an adverse event within six hours had an ECG with evidence of at least one hyperkalemic abnormality (Table 2). QRS prolongation (n=22) and bradycardia of less than 50 bpm (n=17) were the most common ECG abnormalities identified. Of the patients with QRS prolongation, the average QRS duration was 152 msec (SD 35 msec, range 116–266 msec). The majority of the hyperkalemic patients with an adverse event had more than one hyperkalemic ECG abnormality (n=24, 86%). Two patients had isolated bradycardia (HR<50), one patient had isolated junctional rhythm, and one patient had isolated QRS prolongation. No short-term adverse events occurred among patients with isolated peaked T waves or isolated PR prolongation as their ECG manifestation of hyperkalemia.

QRS prolongation had a statistically significant association with short-term adverse events (RR 4.74, 95% CI [2.01–11.15]), as did the presence of junctional rhythm (RR 7.46, 95% CI [5.28–11.13]). Additionally, bradycardia (HR<50 bpm) had a strong positive association with short-term adverse event (RR 12.29, 95%CI [6.69–22.57]) All patients with a ventricular escape rhythm (n=4) developed a short-term adverse event. There was no statistically significant correlation between peaked T waves and short-term adverse events (RR 0.77, 95% CI [0.35–1.70]). Analysis of the association of PR prolongation and adverse events was limited because the majority of the patients who had a short-term adverse event were in a non-sinus rhythm (junctional rhythm n=11, ventricular escape rhythm n=4, atrial fibrillation n=6, 2nd degree heart block n=1). Of the six patients with short-term adverse events who could have a PR interval measured, three patients had PR prolongation.

We calculated the interrater reliability with a kappa value of 1.0 for PR prolongation and QRS prolongation; 0.662 for peaked T waves, 0.716 for rhythm analysis, 0.870 for type of block, and 0.822 for “any abnormality suggestive of hyperkalemia.” The interrater reliability for the presence or absence of a short-term adverse event was strong (kappa 0.870).

## DISCUSSION

This paper is the largest study to date to report the relationship of specific ECG abnormalities to short-term adverse events in patients with severe hyperkalemia (K^+^≥6.5 mEq/L). Short-term adverse events occurred in 15% of patients (95% CI [10.4%–20.7%]). All patients who experienced a short-term adverse event had a preceding ECG that demonstrated at least one hyperkalemic abnormality. An increased likelihood of short-term adverse event was found for hyperkalemic patients whose ECG demonstrated QRS prolongation, bradycardia (HR<50), and/or junctional rhythm.

Previous research has demonstrated that ECG abnormalities and adverse events typically occur in hemodialysis patients at higher serum K^+^ levels than those with preserved renal function.^9^,^19^ In our study, there was no statistical difference between the frequency of hyperkalemic ECG abnormalities and adverse events in established hemodialysis patients and non-dialysis patients. We suggest that this lack of distinction between the two groups derives from the high rate of severe renal impairment (81%) in our study’s non-dialysis patients.

To our knowledge, prior to our publication only two other studies have reported the relationship between ECG findings of hyperkalemia and the development of adverse events.^11^,^16^ Our study methods were designed to minimize limitations in two similar designs.

In An’s study of patients with K^+^≥6.5 mEq/L, ECG findings were correlated with survival to hospital discharge.^16^ In comparison with our study, An’s study did not focus on the use of the ECG for risk stratification of hyperkalemic patients. The most common ECG finding of hyperkalemia was “asystole or pulseless electrical activity,” a reflection of the fact that 20% of the hyperkalemic patients were diagnosed at time of cardiac arrest. In addition, the time from ECG to death was not reported. A significant lapse of time between the ECG and death is suggested by the report that almost half (47%) of patients were not hyperkalemic at the time of their death.

In Montague’s study of 90 patients with K≥6.0 mEq/L, 14 patients experienced arrhythmia or cardiac arrest.^11^ Fewer than half of the patients with arrhythmia or cardiac arrest were noted to have new T-wave peaking or symmetry. Montague’s finding is consistent with our observation that only 25% of patients with short-term adverse events had peaked T waves. However, this study did not discuss the presence or absence of other hyperkalemic ECG manifestations (such as bradycardia, junctional rhythm, PR prolongation, QRS prolongation) in the study population.

In contrast to An and Montague’s methods, no patients in this study experienced a cardiac arrest prior to or during the performance of the ECG. We examined multiple ECG manifestations of hyperkalemia. All adverse events occurred within six hours of the ECG, with a median time from ECG to adverse event of 47 minutes.

In our study, all patients who experienced a short-term adverse event had a preceding ECG that demonstrated hyperkalemic abnormality (100%, 95% CI [85.7–100%]). In fact, the majority of the hyperkalemic patients with a short-term adverse event had more than one hyperkalemic ECG abnormality (86%). However, the small number of adverse events in our study resulted in CIs that were too broad to conclude that hyperkalemia patients without ECG abnormalities do not have short-term adverse events.

Three quarters of patients with short-term adverse events did not have peaked T waves, and there was no statistically significant correlation between the presence of peaked T waves and the development of a short-term adverse events. These findings contradict classic teaching. Texts and papers tend to emphasize peaked T waves as the ECG manifestation of hyperkalemia in their illustrations and research design.^11^,^17^–^19^ In contrast, our study identified QRS prolongation (RR 4.74, 95% CI [2.01–11.15]), junctional rhythm (RR 7.46, 95% [5.28–11.13]), and bradycardia of less than 50 bpm (RR 12.29, 95% CI [6.69–22.57]) as the ECG manifestations of hyperkalemia associated with short-term adverse events.

Interestingly, all adverse events in our study occurred prior to treatment with calcium, and all but one occurred prior to K^+^-lowering intervention. There was no significant difference in time to treatment between patients with or without adverse events. Rather, adverse events occurred either prior to the laboratory notification of hyperkalemia (n=16, 59%) or shortly after the laboratory notification of hyperkalemia (mean 36 min; SD 19 min). One potential application of our study results would be the use of the ECG for early identification of patients who are at higher risk of adverse events. These patients could then be prioritized to rapid treatment (either empirically if clinical suspicion for hyperkalemia is high or after laboratory notification if hyperkalemia was not clinically suspected).

Our findings suggest that the ECG is a useful tool in the stratification of hyperkalemic patients into higher and lower risk groups. This study is the first step in creating a predictive tool for the use of the ECG to identify which hyperkalemic patients are at risk for adverse events.

## LIMITATIONS

The lack of racial/ethnic diversity in our study sample (94% White) may limit the applicability of our findings to more diverse populations.

Severely hyperkalemic patients frequently have additional metabolic abnormalities and these can also affect the ECG. Concurrent metabolic disturbances thought to worsen the ECG manifestations of hyperkalemia (hypocalcemia, hyponatremia, acidemia) were more common in our study than metabolic disturbances thought to lessen the ECG manifestations of hyperkalemia (hypercalcemia, hypernatremia, alkalemia).^2^ These patients’ ECGs can also be affected by underlying cardiac disease. We performed comparison to previous ECG to decrease the effect of baseline ECG abnormalities. Previous ECG was unavailable in 35% of patients. However, we observed no difference between the frequency of hyperkalemic ECG abnormality between patients with or without a previous ECG.

While the ECG readers were blinded to the study methods and objective, the high number of ECGs with hyperkalemic abnormalities, along with a lack of non-hyperkalemic controls, could have led the ECG readers to suspect that the study population contained patients with hyperkalemia.

Our definition of symptomatic bradycardia required both treatment and symptoms. Symptomatic bradycardia may have been underestimated because symptoms may have been present but not recorded in the medical record. All patients treated with atropine, epinephrine, dopamine and/or pacing had recorded symptoms and were classified as symptomatic bradycardia. Four patients were identified who were treated with calcium and had a documented HR of <50bpm within six hours, but were asymptomatic and therefore not classified as an adverse event. All four of these patients had an ECG with hyperkalemic abnormalities.

Patients may have had both severe hyperkalemia and additional acute medical illnesses. The influence of concurrent acute medical illness on the occurrence of adverse events in this study is unknown. However, the majority of patients who experienced an adverse event improved with calcium treatment, suggesting that hyperkalemia was the primary etiology.

Almost all patients (95%) received treatment and the timing and type of treatment was not standardized. Treatment differences had the potential to confound the associations between specific ECG abnormalities and adverse events. However, this was not observed. All adverse events occurred prior to treatment with calcium, and all but one occurred prior to K^+^-lowering intervention. There was no significant difference in time to treatment between patients with or without adverse event, nor for each particular ECG finding. Time to treatment was consistent with previous study of hyperkalemia treatment practices.^8^

## CONCLUSION

Our findings support the use of the ECG in the risk stratification of patients with severe hyperkalemia. All hyperkalemic patients in our sample who experienced a short-term adverse event had a preceding ECG that demonstrated at least one hyperkalemic abnormality. An increased likelihood of short-term adverse event was found for hyperkalemic patients whose ECG demonstrated QRS prolongation, bradycardia (HR<50), and/or junctional rhythm. These data could be used to create a predictive tool to identify which hyperkalemic patients are at risk for adverse events based on ECG findings.

## Supplementary Information



## Figures and Tables

**Table 1:** Demographics and laboratory results of patients with severe hyperkalemia (K^+^≥6.5 mEq/L).

Patient characteristic	N=188
Demographics	
Age, mean (SD), y	68 (16)
Gender, n (%) male	102 (54)
Race, n (%)	
White	177 (94)
Black	4 (2)
Hispanic	1 (0.5)
Other	6 (3)
Laboratory values, mean (SD)	
Potassium level, mEq/L	7.1 (0.6)
Sodium level, mEq/L	135 (6)
Calcium level, mg/dL*	9.0 (1.0)
Bicarbonate level, mEq/L	19 (9)
Estimated glomerular filtration rate, n, (%)†	
<15 mL/min/1.73m2	94 (50)
15–29 mL/min/1.73m2	64 (34)
30–59 mL/min/1.73m2	28 (15)
60–89 mL/min/1.73m2	2 (1)
≥90 mL/min/1.73m2	0 (0)
Established Hemodialysis	32 (17)

*SD*, standard deviation.

* Calcium level was not measured in 26 events (14%).

† Estimated glomerular filtration rate was calculated using the MDRD study equation.

**Table 2:** Electrocardiographic (ECG) findings in patients with severe hyperkalemia.

Characteristic	No adverse event (n=160,%)	Adverse event (n=28,%)	Total (n=188,%)	Relative risk for adverse event (95% CI)
Any ECG abnormality suggestive of hyperkalemia	106 (66)	28 (100)	134 (71)	‡
Peaked T waves	50 (31)	7 (25)	57 (30)	0.77 (0.35–1.70)
PR prolongation†	25 (18)	3 (50)	28 (20)	4.11 (0.88–19.28)
QRS prolongation	60 (38)	22 (79)	82 (43)	4.74 (2.01–11.15)*
Mild QRS prolongation (111–119 msec)	13 (8)	2 (7)	15 (8)	
Left bundle branch block	8 (5)	3 (11)	11 (6)	
Right bundle branch block	17 (11)	10 (36)	27 (14)	
Nonspecific intraventricular conduction delay	22 (14)	7 (25)	29 (15)	
Bradycardia (HR<50 bpm)	4 (3)	17 (61)	21 (11)	12.29(6.69–22.57)*
Junctional rhythm	4 (3)	11 (39)	15 (8)	7.46 (4.32–12.87)*
Ventricular escape rhythm	0 (0)	4 (14)	4 (2)	7.67 (5.28–11.13)*
Ventricular tachycardia	NA	2 (7)	2 (1)	NA
2nd Degree heart block	0 (0)	1 (4)	1 (0.5)	6.92 (4.88–9.82)
3rd Degree heart block	0 (0)	0 (0)	0 (0)	NA

Patients may have had more than one hyperkalemic ECG abnormality.

† PR interval measured in 143 episodes (137 episodes without adverse event and 6 episodes with adverse event). PR interval was unable to be measured in 45 episodes due to non-sinus rhythm.

‡ Relative risk unable to be calculated as no adverse events occurred in patients without ECG abnormality suggestive of hyperkalemia

* p<0.05

## References

[1] Pfenning C, Solovis C, Marx J, Hockberge R, Walls R (2014). Electrolyte Disorders. Rosen’s Emergency Medicine Concepts and Clinical Practice.

[2] Weisberg L (2008). Management of severe hyperkalemia. Crit Care Med.

[3] Alfonzo A, Soar J, MacTier R Clinical practice guidelines treatment of acute hyperkalemia in adults.

[4] Medford-Davis K, Rafique Z (2014). Derangements of potassium. Emerg Med Clin North Am.

[5] Trulhar A, Deakin C, Soar J (2015). European Resuscitation Council Guidelines for Resuscitation 2015 Section 4. Cardiac arrest in special circumstance. Resuscitation.

[6] Acker C, Johnson J, Palevsky P (1998). Hyperkalemia in hospitalized patients: causes, adequacy of treatment, and results of an attempt to improve physician compliance with published therapy guidelines. Arch Intern Med.

[7] Fordjour K, Walton T, Doarn J (2014). Management of hyperkalemia in hospitalized patients. Am J Med Sci.

[8] Freeman K, Feldman J, Mitchell P (2008). Effects of presentation and electrocardiogram on time to treatment of hyperkalemia. Acad Emerg Med.

[9] Khattak H, Khalid S, Manzoor K (2014). Recurrent life-threatening hyperkalemia without typical electrocardiographic changes. J Electrocardiol.

[10] Szerlip H, Weiss J, Singer I (1986). Profound hyperkalemia without electrocardiographic manifestations. Am J Kidney Dis.

[11] Montague B, Ouellette J, Buller G (2008). Retrospective review of the frequency of ECG changes in hyperkalemia. Clin J Am Soc Nephrol.

[12] Weingart S Podcast 32-Treatment of severe hyperkalemia.

[13] Welch A, Maroz N, Wingo C (2013). Hyperkalemia: getting to the heart of the matter. Nephrol Dial Transplant.

[14] Burns E Hyperkalemia. Life in the FastLane.

[15] Rossignol P, Legrand M, Kosiborod M (2016). Emergency management of severe hyperkalemia: guideline for best practice and opportunities for the future. Pharmacol Res.

[16] An J, Lee J, Jeon HJ (2012). Severe hyperkalemia requiring hospitalization: predictors of mortality. Crit Care.

[17] Chon S, Kwak Y, SSH (2013). Severe hyperkalemia can be detected immediately by quantitative electrocardiography and clinical history in patients with symptomatic or extreme bradycardia: a retrospective cross-sectional study. J Crit Care.

[18] Green D, Green H, New D (2013). The clinical significance of hyperkalaemia-associated repolarization abnormalities in end-stage renal disease. Nephrol Dial Transplant.

[19] McCullough P, Beaver T, Bennett-Guerrero E (2014). Acute and chronic cardiovascular effects of hyperkalemia: new insights into prevention and clinical management. Rev Cardiovasc Med.

