# Supplementary material for: Severe Hyperkalemia: Can the Electrocardiogram Risk Stratify for Short-term Adverse Events?
Source: West J Emerg Med. 2017 Jul 10;18(5):963–71. doi: 10.5811/westjem.2017.6.33033 (PMC5576635; doi:10.5811/westjem.2017.6.33033)
Supplement: Supplementary file 1 [file wjem-18-963-s001.docx]

Appendix. Electrocardiographic (ECG) Findings in Patients with Severe Hyperkalemia with a Previous ECG Available (n=123, 65% of study population).

| Characteristic | | | No Adverse Event (n=105, %) | | Adverse event (n=18, %) | Total (n=123,%) | | Relative Risk for Adverse Event  (95% CI) | |  |  |  |
| --- | --- | --- | --- | --- | --- | --- | --- | --- | --- | --- | --- | --- |
| Any ECG abnormality suggestive of hyperkalemia | | | 67 (64) | | 18 (100) | 85 (69) | | ‡ | |  |  |  |
| Peaked T waves | | | 38 (30) | | 2 (11) | 40 (33) | | 0.26 (0.06-1.07) | |  |  |  |
| PR prolongation† (out of 97) | | | 16 (17) | | 2 (50) | 18 (15) | | 4.39 (0.66-29.1) | |  |  |  |
| QRS prolongation | | | 30 (29) | | 12 (67) | 42 (34) | | 3.86 (1.56-9.54)* | |  |  |  |
| Bradycardia (HR<50 bpm) | | | 4 (4) | | 12 (67) | 16 (13) | | 13.38 (5.85-30.59)* | |  |  |  |
| Junctional Rhythm | | | 2 (2) | | 7 (39) | 9 (7) | | 8.06 (4.16-15.62)* | |  |  |  |
| Ventricular Escape Rhythm | | | 0 (0) | | 2 (11) | 2 (2) | | 7.56 (4.79-11.94)* | |  |  |  |
| Ventricular Tachycardia | | | NA | | 1 (6) | 1 (1) | | NA | |  |  |  |
| 2^nd^ Degree Heart Block | | | 0 (0) | | 1 (6) | 1 (1) | | 7.17 (4.62-11.15) | |  |  |  |
| 3^rd^ Degree Heart Block | | | 0 (0) | | 0 (0) | 0 (0) | | NA | |  |  |  |
|  | |  | |  | | |  | |  | | |  |
|  | Patients may have had more than one hyperkalemic ECG abnormality. Relative risk for adverse events was comparable to those for the full study population.  †PR interval measured in 97 episodes (93 episodes without adverse event and 4 episodes with adverse event). PR interval was unable to be measured in 26 episodes due to nonsinus rhythm.  ‡Relative risk unable to be calculated as no adverse events occurred in patients without ECG abnormality suggestive of hyperkalemia  *p<0.05 | | | | | | | | | |  |  |
